# The Effect of Oral Propranolol versus Oral Corticosteroids in Management of Pediatric Hemangiomas

**Published:** 2018-01

**Authors:** Adil Ali, Umme Aiman, Mohd Azam Haseen, Mohd Altaf Mir, Imran Ghani, Ragya Bharadwaj, Mohd Yaseen

**Affiliations:** Sir Syed Nagar, JNMCH, AMU, Aligarh, India

**Keywords:** Pediatric hemangiomas, Propranolol, Corticosteroids

## Abstract

**BACKGROUND:**

Hemangiomas are the most common benign tumors of infancy. This study evaluated the efficacy of oral propranolol comparing to oral steroids in management of pediatric hemangiomas.

**METHODS:**

In North India from January 2012 to January 2015, sixty children <6 years old with superficial hemangiomas were divided into 2 groups; oral propranolol vs. oral prednisolone. All participants were assessed for electrocardiogram, heart rate, blood pressure and sugar and initial therapy was started using 1 mg/kg and in absence of adverse effects, 2 mg/kg was administered after 2 weeks. The hemangioma Activity Score (HAS) was used for scoring and patients were followed up for 6 months.

**RESULTS:**

The propranolol group mostly showed early response to the drug and needed the drug for less time compared to corticosteroid group. In propranolol group, 16.5%, 23% and 59% needed the drug to be continued for 8-12, 4-8 and 4 months. In corticosteroid group, the therapy was continued for 8-12, and 4-8 months in 76.8% and 16.5% and in 6.6% was stopped within 4 months. In propranolol group, the response was 70% compared to 40% in other group. The mean HAS decreased significantly in propranolol group when compared to steroid group. Three patients on prednisolone developed Cushingoid features, while 1 patient in propranolol group had mild flue like symptoms.

**CONCLUSION:**

Two mg/kg of oral propranolol significantly decreased HAS, when compared to oral prednisolone, with good parent satisfaction, minimal adverse effects and no recurrence/relapse of hemangiomas after a follow up period of 6 months.

## INTRODUCTION

Haemangiomas are the most common benign tumors of infancy.^1^ Their prevalence has been estimated at 10%, however in premature infants it can be as high as 20-30%.^2^ It is more common in girls and is seen predominantly on face and head neck region,^2^ however it can occur anywhere in body including airway.^3^ Infantile Hemangiomas (IH) are benign tumors that are usually not present at birth but instead are noted within the first few weeks of life. Although most lesions proliferate and then involute with minimal consequence, a significant minority can be disfiguring, functionally significant, or, rarely, life-threatening.^4^

Leaute-Labreze *et al.* reported incidental decrease in size of infantile haemangiomas on propranolol with dramatic results.^5^ Potential explanations for this effect given by them were vasoconstriction, which is immediately visible as a change in color, associated with a palpable tissue softening. Other included suggestions are a down-regulation of angiogenetic factors such as VEGF and bFGF and an up-regulation of apoptosis of capillary endothelial cells.^5^ There are also data published which indicate a selective role of propranolol in inhibiting the expression of MMP-9 (angiogenic and extracellular matrix degrading enzyme) and HBMEC (human brain microvascular endothelial cells).^6^

Janmohamed *et al.* developed a simple system called the Haemangioma Activity Score (HAS) for scoring the (disease) proliferative activity of haemangiomas.^7^ A number of treatment options are available for the treatment of these tumors like intralesional or systemic steroids, interferon alpha, vincristine, bleomycin, pulsed dye lasers, etc.^8^ Surgical treatment is sometimes required if hemangiomas fail to involute causing aesthetically significant disfigurement associated with or without psychological and physical distress.^9^ In this study, we have compared safety and efficacy of oral propranolol versus oral corticosteroid in pediatric hemangiomas in Indian population.

## MATERIALS AND METHODS

While there are several studies from developed countries on safety and efficacy of oral propranolol in management of hemangioma,^5^ but only a few have been reported from Indian subcontinent. We wanted to test the efficacy and safety of propranolol in Indian patients as there can be variations in response to the same drug in different populations, and also wanted to measure the aesthetic improvement of the lesions to the two modalities of treatments. Our study is non randomized, open label, parallel arm, experimental study; ethical clearance was taken from Institutional Ethics Committee. The Haemangioma Activity Score (HAS) developed by Janmohamed *et al.*^7 ^(Table 1) was used for scoring the activity of the haemangioma to the two treatment options.

**Table 1:** Haemangioma Activity Score.

**Date**	
Deep swelling:	Tense HOI (6) ‘Neutral’ HOI at t=0 or less than 50% reduction at follow up (4) ≥50% reduction at follow up (2) No more swelling at follow up (0)
Bright red/shining red HOI (5) OR Bright red edge (4)	
Matt red/reddish-purple HOI/ matt red edge (3)	
Blue HOI or blue shining through in deep HOI (2)	
Grey HOI (1)	
Skin coloured after activity (0)	
Total score	
Number of items scored	
Preliminary HAS=total score/number of items scored	
Ulcer ≤1 cm^2^ (+0.5) Ulcer 1-25 cm^2^ (+1) Ulcer ≥25 cm^2^ (+2)	
HAS=Preliminary HAS+ulcer score	

The efficacy of oral corticosteroid and oral propranolol in patients of pediatric hemangiomas was compared by assessing the decrease in the HAS at 0, 1, 4, 8, 24, 32 and 48 weeks after initiation of therapy by comparing serial photographs. We also noted the complications of oral propranolol and oral corticosteroid (prednisolone) in treatment of pediatric haemangiomas during the follow up schedules and parent satisfaction to the treatment. 

Sixty children were included in this study, all children were <6 years of age and had superficial haemangiomas. Contraindications to therapy like asthma, heart block or other congenital heart disease were ruled out. Patients with multicentric hemangiomas or intracavitary lesions were excluded from study. After allocating patients into 2 groups; oral propranolol vs. oral prednisolone, all participants were admitted for 24 hours and base line electrocardiogram, heart rate, blood pressure and blood sugar were recorded. 

In both the groups, patients were initially started therapy with a dose of 1 mg/kg body weight and once no adverse effects were seen the dose was increased to 2 mg/kg body weight during second week of treatment. Standard photographs were taken at baseline (t0), 1 week (t1), 4 weeks (t4), 8 weeks (t8), 32 weeks (t32) and 48 weeks (t48) of the follow up periods and the HAS score was calculated at each visit. Therapy was stopped when more than 75% response to treatment was achieved or once treatment schedule had completed 48 weeks or if there were severe side effects to therapy. A ‘good response’ was defined as more than 50% reduction in HAS, a ‘favorable response’ was defined as a 30% to 50% reduction in HAS, a ‘non-responder’ was defined when HAS decreased less than 30%. 

Baseline characteristics were analyzed by descriptive statistics. Continuous data was summarized as mean and standard deviation. Categorical data was summarized as numbers and percentages. For the primary analysis, the difference of HAS between two groups was analyzed by linear mixed model. Complications between two groups were analyzed using t-test or Chi Square test as appropriated. All analyses were performed according to the intention-to-treat principle. Missing values, if present, were corrected with last observation carry forward.

## RESULTS

A total of 60 patients were included in this study, 30 patients (50%) were allocated to oral propranolol group (study group) and 30 patients (50%) were allocated to oral prednisolone group (control group). There was female preponderance in each group with a male: female ratio of 2:3. The most common age of presentation was <12 months (58%) and 70% of the lesions were located in the head, neck and face region. On further breakdown, we found that in head and neck region, lesions were most commonly concentrated around cheeks and pre auricular region (Table 2).

**Table 2:** Correlation of response to propranolol with age of presentation.

**Age at presentation**	**Good responders (21)**	**Non-responders (5)**
<6 months	11 (52.4%)	--
6-12 months	7 (33.3%)	1 (20%)
>12 months	3 (14.3%)	4 (80%)

**Fig. 1:**
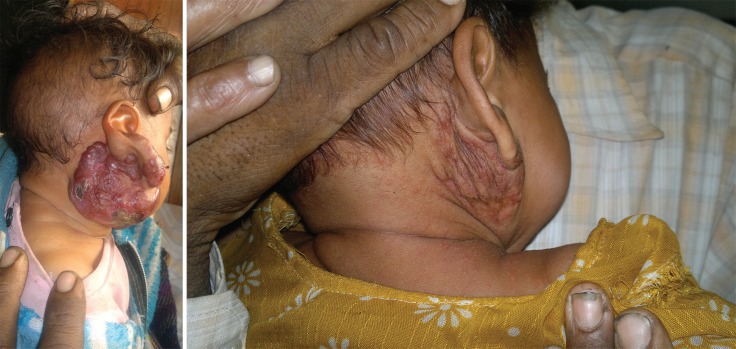
**Left. **Pretreatment. 5x7x4 cm lesion on the right infraauricular region. **Right. **Post-treatment at 3 months.

**Fig. 2:**
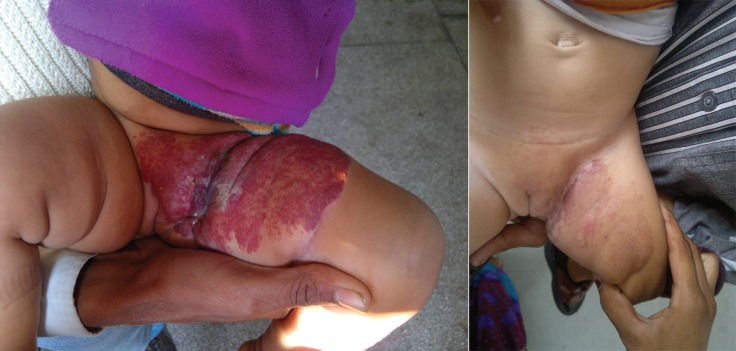
**Left. **pretreatment 7x8 cm lesion left groin. **Right. **Post treatment at 3 months.

**Fig. 3:**
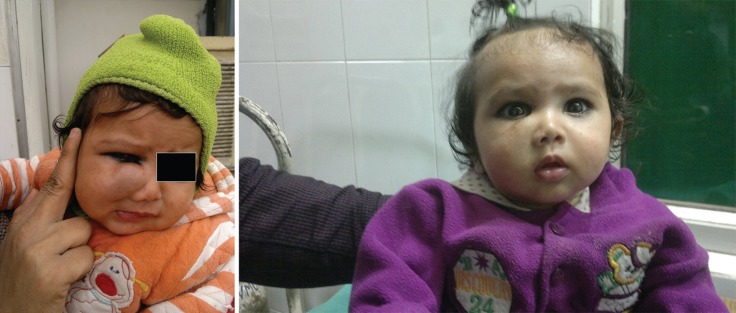
**Left. **Pretreatment 3x2 cm lesion right infra orbital region. **Right. **Posttreatment at 6 months.

**Fig. 4:**
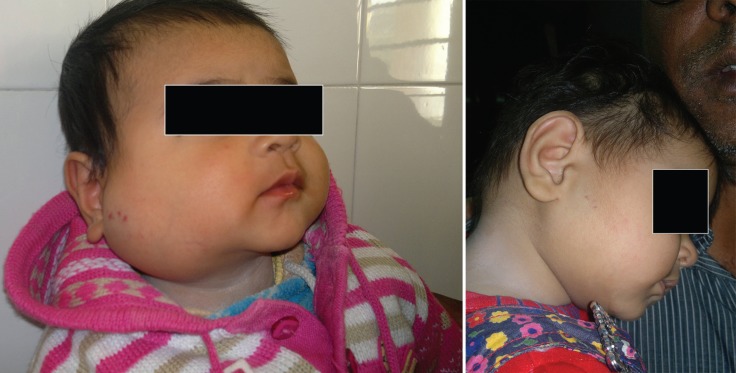
**Left. **Pretreatment lesion 5x7 cm right cheek. **Right. **Posttreatment.

**Fig. 5:**
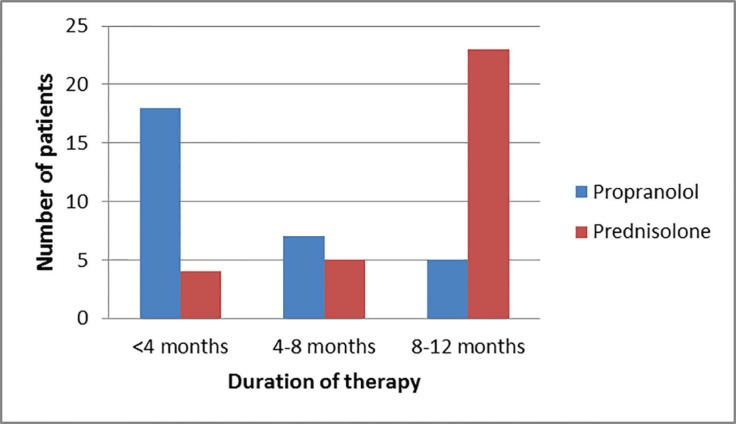
Duration of therapy.

In our study, the patients who were on propranolol mostly showed early response to the drug and needed the drug for less time as compared to patients taking corticosteroids. In the propranolol group, only 16.5% patients needed the drug to be continued for 8-12 months, while in 23% it was continued for 4-8months and 59% children showed a response within 4 months. While in corticosteroid group, the therapy was continued for 8-12 months in 76.8% patients, 4-8 months in 16.5% patients and in 6.6% of patients, therapy had to be stopped within 4 months, mainly due to complications of prednisolone treatment (Figure 1-5).

**Fig. 6:**
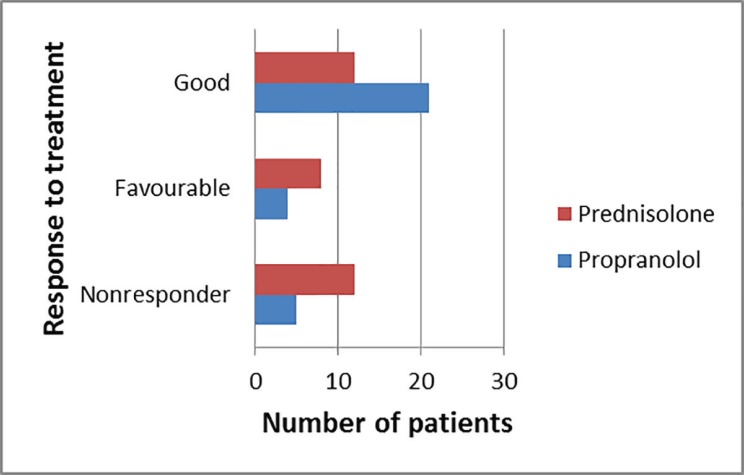
Response of patients to treatment.

In the propranolol group, the response was ‘good’ in majority of cases (70%) as compared to 40% in steroid group (Figure 6). In steroid group, 33.3% of patients were non responders while in propranolol group only 16.7% were non-responders. The difference between the groups was statistically significant (*p*=0.028). When we split the patients taking propranolol on the basis of their age of presentation, we found that the children who presented in infancy (85% responders) were more likely to show response to propranolol than those who present later (Table 1). This was found to be statistically significant (*p*=<0.001). Of the total 15 non responders (10 from prednisolone and 5 from propranolol group), no switch over was made; 8 children needed surgical intervention and in 7 injection sclerotherapy was given. 

**Fig. 7:**
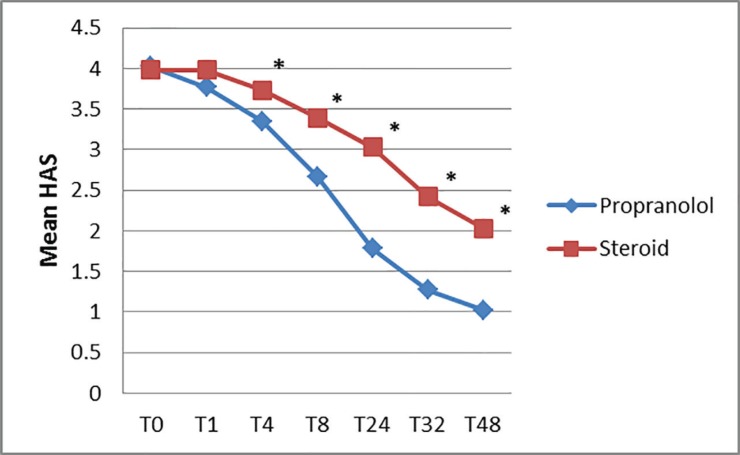
Comparison of mean HAS (Haemangioma Activity Score) between propranolol and prednisolone group with duration of follow up (*p<0.01)

HAS was measured in all patients at t0, t1, t4, t8, t24, t32 and t48 (Figure 7). The mean HAS was similar in propranolol and prednisolone group at t0, but the HAS in propranolol group started decreasing rapidly and became statistically significant at 4 weeks. Thereafter, HAS score in propranolol remained statistically less at all time intervals and the mean HAS decreased significantly in the propranolol group (from 4.02 to 1.02) when compared to the steroid group (from 3.98 to 2.03). 

**Fig. 8:**
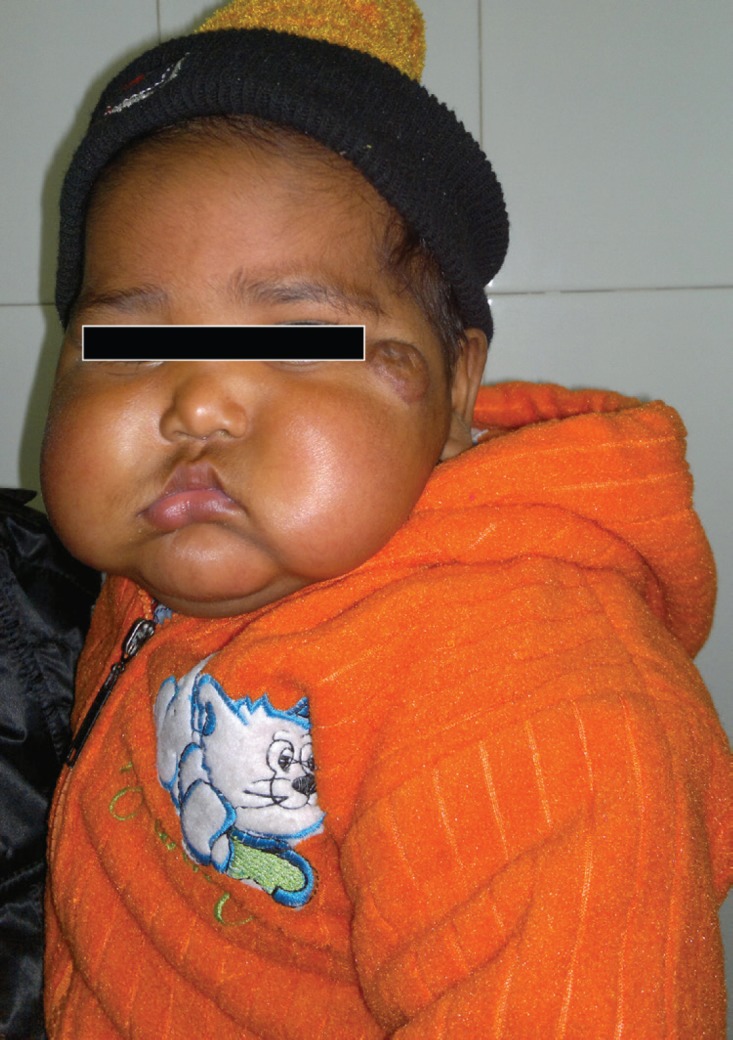
Cushingoid features on treatment with corticosteroids.

Parent satisfaction was higher in propranolol group (77% vs. 40%) owing to good response to treatment (70% in propranolol group vs. 40% in prednisolone group) and less side effects (6.7% vs. 12.4%, respectively). Three patients (10%) on oral prednisolone developed Cushingoid features (Figure 8) and hence their treatment had to be stopped within 3 months, while 1 patient (3.33%) in the oral propranolol group had mild flue like symptoms and no other patient in the propranolol group reported any other symptoms.

## DISCUSSION

Infantile haemangioma (IH) is the most frequent childhood tumor. Although it is benign and self-limiting, severe complications can arise due to fast growth. It is difficult to pin point one event in pathogenesis of hemangioma, rather there are several mechanisms which act together, although local hypoxia seems to be the most important factor. Three main hypotheses have been proposed, namely (i) the theory of tissue hypoxia, (ii) the theory of embolization of placental endothelial cells, and (iii) the theory of increased angiogenic and vasculogenic activity.^10^ Though most of the cases occur in head and neck region, it can occur in airway, perineum and even body cavities.^3^^,^^11^^,^^12^ There is confusion whether infantile hemangioma is an isolated entity or a spectrum of vascular anomalies including rapidly involuting congenital hemangioma (RICH ) and Non-involuting congenital hemangioma (NICH). RICH and NICH have similarities in appearance, location, size, equal sex ratio, and both have overlapping radiologic and histologic features with infantile hemangioma. However, neither type of congenital tumor has immunostains for glucose transporter-1 protein, a marker of infantile hemangioma.^13^

Prior to use of propranolol for management of hemangiomas, corticosteroids were the first line of treatment;^14^ other options included interferon alfa^15^ and vincristine.^16^ Propranolol hydrochloride, a nonselective β-adrenergic blocking agent, has been used for years in infants and children with congenital heart disease, arrhythmias, hypertension, thryotoxicosis, migraines, and behavioral disorders. It has recently been used in the treatment of infantile hemangiomas (IHs) after growth arrest of an infant’s hemangioma was incidentally noted when propranolol was started for obstructive hypertrophic myocardiopathy by Leaute-Labreze *et al.*^17^

These authors initiated propranolol therapy to 2 children who had severe or disfiguring capillary haemangiomas on a dose of 2 mg/kg, after encouraging results they further gave the drug to another 9 patients. In all patients, after 24 hours of initiation of treatment, they observed a change in the haemangioma from intense red to purple. After these initial changes the haemangiomas continued to improve until they were nearly flat, with residual skin telengectasias.^5^ The therapeutic effect of propranolol on infantile capillary hemangiomas is because of vasoconstriction leading to immediate improvement and decreased expression of VEGF and bFGF genes through the down-regulation of the RAF–mitogen-activated protein kinase pathway^18^ and the triggering of apoptosis of capillary endothelial cells^19^ leading to long term improvement. 

Holmes *et al.* prospectively assessed the safety and efficacy of propranolol for problematic haemangiomas. Propranolol in a dose of 3 mg/kg was given to 31 consecutive patients who had rapidly proliferating infantile haemangiomas with functional impairment or cosmetic disfigurement. A rapid halt in haemangioma proliferation was seen in 100% patients and significant reduction in 87% of patients.^20^ Leaute-Labreze *et al.* performed a multicenter, randomized, double-blind, adaptive, phase 2–3 trial assessing the efficacy and safety of a pediatric-specific oral propranolol solution in infants 1 to 5 months of age with proliferating infantile hemangioma requiring systemic therapy.^21^

Of 460 infants who underwent randomization, 456 received treatment. On the basis of an interim analysis of the first 188 patients who completed 24 weeks of trial treatment, the regimen of 3 mg of propranolol per kilogram per day for 6 months was selected for the final efficacy analysis. The frequency of successful treatment was higher with this regimen than with placebo (60% vs. 4%, *p*<0.001). A total of 88% of patients who received the selected propranolol regimen showed improvement by week 5, versus 5% of patients who received placebo.^21^

In our study, also propranolol has been found to be very safe at dose of 2 mg/kg except for mild side effects in two patients (6.7%). In our study, 84% patients showed response to propranolol treatment of which 70% (21 patients) had complete resolution. Once we analyzed the non-responders to propranolol, we found that that those who presented after 1 year of age are more likely to show less response (80 % non responders in >1 yr of age Vs 20 % non-responders in <1 year of age), probably because of cessation of proliferative activity of tumor beyond infancy. 

While many authors believe good response is expected when the therapy is started within the first 12 months of life,^22^ there are studies contrary to this, which state that the involution seems not to depend on the location of the hemangioma or the age at initiation of therapy.^23^ Razon *et al*. demonstrated that the involutive phase of IH involves high apoptosis and low proliferation of endothelial and hence efficacy of propranolol will decrease as proliferation has decreased after 1 year of age.^24^

We did not observe any severe adverse effect in our patients effects except for transient flu like illness and mild brochospasm in one patient each which resolved by itself. Though safety of propranolol has been established by large randomized controlled trials,^25^ however, still there are few case reports of severe side effects with use of propranolol like hypoglycemia, hypotension, bronchial asthma, flu like illness, somnolence, gastroesophageal reflux, respiratory syncytial virus exacerbation and rash.^26^^-^^28^

An important way to avoid complications with propranolol therapy is to start it at low dose of 1 mg/kg and gradually escalate it to 2-3 mg/kg. Basic investigations (like ECG, heart rate, blood pressure and blood sugar) should be done at beginning and repeated at regular intervals to monitor therapy. Other oral beta-blockers such as atenolol, nadolol and acebutalol have also been used in hemangioma, however they are less studied, but may offer similar efficacy.^25^^,^^29^

No trials have compared safety and efficacy of different β-blockers in management of hemangiomas. Topical 0.5% timolol maleate has been recently tried and shown good results in superficial haemangiomas with the added advantage of very minimal systemic absorption. There are also case reports of use of ACE inhibitors for IH with good results.^30^ As has been mentioned in several articles, there is a chance that IH successfully treated with propranolol may recur 0–6 months after therapy withdrawal. The frequency of recurrences in patients treated with propranolol has not been well-characterized. In the series of cases reported by Sans *et al.*,^31^ two of 25 patients (8%) had recurrences after treatment withdrawal. The overall response to retreatment with propranolol was satisfactory. These relapses occurred before the age of 11 months, which might mean that the treatment was withdrawn before the proliferative phase of the haemangioma was over. There was no reported recurrence of haemangioma on stopping therapy in our study, even after a follow up of 6 months. 

We conclude that oral propranolol in a dose of 2 mg/kg significantly decrease the HAS, when compared to oral prednisolone, with good parent satisfaction, minimal adverse effects and no recurrence/relapse of haemangiomas after a follow up period of 6 months following completion of therapy. Starting propranolol therapy in proliferative phase of hemangioma yields better results, than when it is started after infancy. However, propranolol for hemangiomas is an off-label-indication and the parents have to be well informed about its side effects. In all, propranolol appears to be an effective treatment for pediatric haemangiomas in the proliferative phase and can be considered as a first-line treatment in hemangiomas when intervention is required.
